# Mach–Zehnder Modulators for Microwave Polarization Measurement in Astronomy Applications

**DOI:** 10.3390/s23146300

**Published:** 2023-07-11

**Authors:** Francisco J. Casas, Guillermo Pascual-Cisneros

**Affiliations:** Instituto de Física de Cantabria (IFCA), Avda. Los Castros s/n, 39005 Santander, Spain; pascual@ifca.unican.es

**Keywords:** instrumentation, Mach–Zehnder modulators, polarization, cosmic microwave background, astronomy

## Abstract

This paper presents a study of the performances of different Mach–Zehnder modulation technologies with applications in microwave polarimeters based on a near-infrared (NIR) frequency up-conversion stage, allowing for optical correlation and signal detection at a wavelength of 1550 nm. Commercial Mach–Zehnder modulators (MZMs) are traditionally implemented using LiNbO_3_ technology, which does not enable integration for the fabrication of MZMs. In this work, we propose the use of an alternative technology based on InP, which allows for integration in the fabrication process. In this way, it is possible to obtain advantages in terms of bandwidth, cost, and size reductions, which yield results that are very interesting for wide-band applications such as microwave instrumentation for the study of the cosmic microwave background (CMB). Here, we describe and compare the modulation performances of different MZMs, with one commercial unit presenting a higher bandwidth than those in previous works, and another three InP integrated units provided by the Fraunhofer Institute for Telecommunications, Heinrich-Hertz-Institute (HHI). Then, these modulators were coupled to a microwave polarimeter demonstrator, which has also been presented previously, to compare the polarization measurement performances of each of the MZMs.

## 1. Introduction

In 1964, a noise-like signal [1] was identified as the cosmic microwave background (CMB) by Penzias and Wilson. As postulated by Gamow, Alpher, and Herman in the late 1940s [2], this radiation turned out to be the footprint remaining from the Big Bang. Since then, many ground-based experiments (e.g., [3,4,5,6,7,8]), balloon-borne investigations (e.g., [9,10]), and space missions [11,12,13] have been dedicated to the characterization of the CMB’s temperature and polarization anisotropies. It is important to note the great advantage of balloons and space missions in terms of sensitivity, mainly due to the absence of atmosphere, which reduces the available frequency range of observation and adds noise, thus affecting the detectors in ground-based experiments. However, the lowest part of the CMB frequency spectrum (practically ranging from 0 GHz to approximately 20 GHz) is particularly interesting for ground-based experiments, which are generally much less expensive. On the one hand, at these frequencies, signals from the sky are dominated by more powerful foregrounds (for instance, synchrotron), requiring far less sensitivity and a lower number of detectors. On the other hand, the lowest frequencies require the implementation of large and heavy waveguide components (antennas, polarizers, etc.), which results in a limiting factor for space instrumentation. Additionally, the superconducting detection technology (Transition Edge Sensors, or TES) that is expected to be used in future CMB space missions (LiteBIRD [14] and PICO [15]) has not yet been proved for this low frequency range. For all these reasons, this work is focused on instrumentation to be applied mainly in ground-based experiments.

In general, CMB observations are a great resource that allow one to test cosmological models and fundamental physical processes due to the extremely weak but distinct features imprinted on the uniform backgrounds of photons from the early universe. 

**Figure 1:**
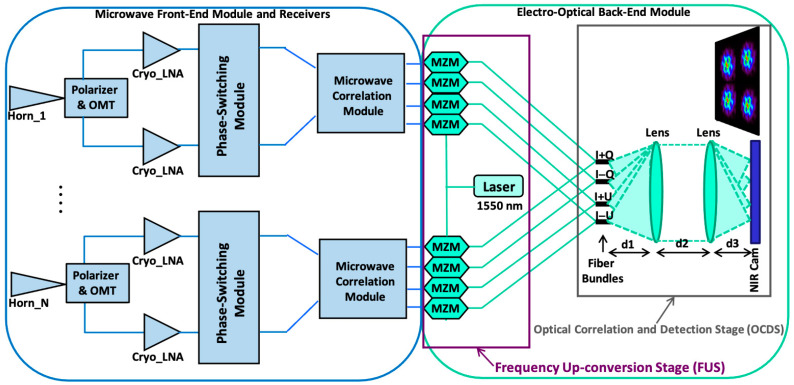
Simplified block diagram of the polarimeter demonstrator with correlation/detection in the near-infrared spectrum (1550 nm). The blue part corresponds to that implemented in microwave technology, while the green one is implemented by means of photonic/optical technology. Reproduced with permission from F. J. Casas, *Sensors*, published by MDPI, 2019 [19].

Today, many radio astronomy instruments have the goal of characterizing both the electric-like (E) and magnetic-like (B) modes of polarization patterns. The standard cosmological model has been validated through E-mode polarization measurements [16], but primordial B-modes of polarization have not yet been detected (see [5,17]). Although they are faint and easily contaminated, they may also reveal crucial information about factors such as inflation, the primordial background of gravitational waves, or galactic and extragalactic magnetic fields. Due to the great interest in these scientific areas, present and future CMB polarization experiments, both on the ground (CMB-S4, [18]) and in space [14,15], are pursuing an unprecedented level of sensitivity. This sensitivity can be achieved mainly through observations with thousands of superconducting detectors. However, as has been stated previously, in order to characterize the foregrounds affecting the lowest part of the CMB frequency spectrum, radiometric receivers based on cryogenic low-noise amplifiers are also commonly used. For these lowest frequencies of the CMB spectrum, in a previous work [19], a 10–20 GHz polarimeter demonstrator was presented, allowing for optical correlation and signal detection at a wavelength of 1550 nm. Each receiver of the polarimeter presented four NIR output signals, allowing for the measurement of the polarization degree and the polarization angle [19,20] independently of one another. In Figure 1, one can see a simplified block diagram of the polarimeter. 

The polarimeter scheme in Figure 1 is composed of a front-end module (FEM) consisting of several microwave receivers and an electro-optical back-end module (EOBEM) with a frequency up-conversion stage (FUS) at the input, connected to an optical correlation and detection stage (OCDS). The detection stage is composed of the EOBEM with the input microwave signals entering the NIR FUS, which is composed of a laser and a set of Mach–Zehnder modulators (MZM), as well as an OCDS implemented in a basic manner with a fiber array, a pair of lenses, and a camera. This scheme was proposed as a solution for the implementation of ultrasensitive large-format interferometers with hundreds of receivers to characterize the polarization B-modes from the lowest frequencies of the CMB spectrum (around 40 GHz in this case, therefore requiring a high number of receivers due to the lower foregrounds and higher CMB signal level). The operation of the proposed polarimeter, as an interferometer, is like that of the Q–U bolometric interferometer for cosmology (QUBIC) [21] that operates using TES at higher frequencies (150 and 220 GHz), where the CMB signal is not affected by low-frequency foregrounds, such as synchrotron. On the other hand, as stated in [19], this scheme can also be applied in direct imaging experiments (for instance, QUIJOTE [20,22] or LSPE–Strip [23]) using the appropriate optical configuration in the OCDS (distances d1, d2 and d3). 

The proposed Instrument was designed to measure the polarization of the microwave radiation from the sky, providing the Stokes polarization parameters [24] of the given signal. To achieve polarization information with good control of the systematics, a polarization modulation component is included in each of the receivers. In this work, they are the phase-switching modules that can be seen in the microwave front-end in Figure 1. In a previous work [25], these modulation modules were implemented in the photonic part of the receivers, using optical voltage-controlled phase sifters. 

In this work, a study of the performances of different Mach–Zehnder modulation technologies is carried out, considering their application to the previously presented [19] microwave polarimeter (see Figure 1). The performances of the modulators used in that work have already been described in [26]. Here, we focus first on a commercial alternative in LiNbO_3_ technology, which offers a higher bandwidth and the possibility of operating with single sideband and suppressed carrier (SSB-SC) modulation. This type of modulation is particularly interesting for the use of the proposed polarimeter as a synthesized-imaging interferometer, since this mode of operation requires the removal of the optical carrier and one of the sidebands from the modulated signal [19]. Another alternative, implemented using InP integrated technology, is then proposed, taking into account the advantages of this technology in terms of bandwidth and potential cost reduction. Two devices provided by the Fraunhofer Institute for Telecommunications, Heinrich Hertz Institute (HHI) have been tested. The first takes advantage of the technology’s integration capabilities and contains two modulators in the same package. The second is a similar unit, but with only one MZM in a chassis similar in size to the integrated version. This last unit was used to ensure that the performance of an integrated InP modulator remained the same as that of a typical commercial unit, but with greater bandwidth and lower cost.

This work is organized as follows: in Section 2, the commercial LiNbO_3_ technology MZM laboratory measurement results are described; in Section 3, the laboratory performance characterization of the InP technology-integrated MZMs is presented; in Section 4, the different MZM alternatives are applied to the implementation of a microwave polarimeter, as presented in [19], showing the performances provided by each alternative in terms of polarization angle measurement and polarization efficiency; finally, in Section 5, general conclusions are drawn.

## 2. LiNbO_3_ Commercial MZMs

For the implementation of the polarimeter presented in [19], a set of commercial LiNbO_3_ technology MZMs were used. These MZMs have a bandwidth of 10 GHz and, as mentioned above, their main characteristics have been presented in [26]. The reduced bandwidth of these MZMs was the main reason for having a polarization efficiency of about 50% [19], instead of a more typical value of about a 90%, which can be found in actual experiments such as QUIJOTE [27]. The obvious way to solve this problem is to use an alternative (and more expensive) MZM that at least provides a higher bandwidth to avoid significant losses in polarization efficiency. In the following, we propose the use of an alternative commercial MZM that solves the bandwidth problem, while offering additional advantages for its application to a polarimeter, such as the one proposed in [19].

### SSB-SC 15 GHz Bandwidth MZM

A good alternative to the MZMs used in [19] is a commercial model from Thorlabs (LN86P–FC [28], now replaced by LNQ4314 [29]), which offers the capability of providing a single sideband with suppressed carrier modulation, while providing about 15 GHz of electro-optical (−3 dB) bandwidth (the data sheet can be downloaded from [28]). As mentioned above, this option is particularly interesting for polarization measurements using a synthetized-image interferometer because of the need to remove both one side band and the laser carrier from the frequency up-converted signal. 

**Figure 2:**
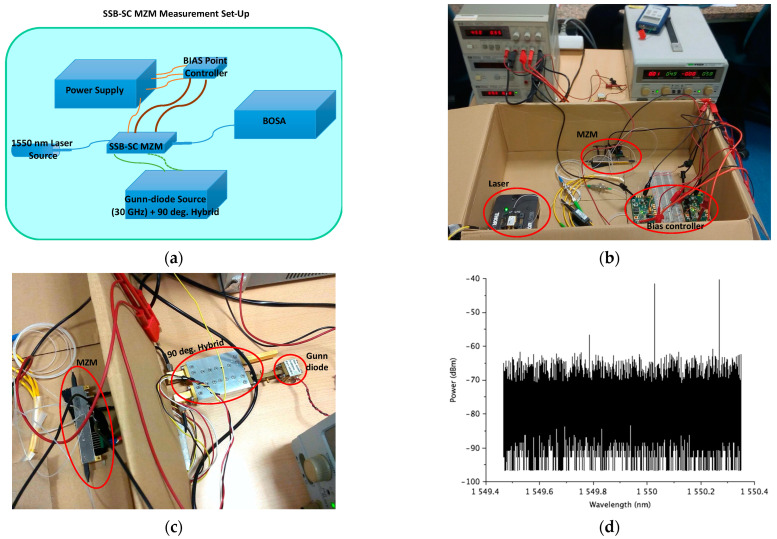
(**a**) Sketch of the measurement set-up mounted in the laboratory; (**b**) picture with the laser (left), the bias point controller (right) and the MZM (center); (**c**) picture with the MZM (left) and the Gunn diode coupled to a waveguide 90-degree hybrid (right); (**d**) modulated signal measured with the optical spectrum analyzer for the MZM bias point: V_RF1_ = 5.1 V, V_RF2_ = 6 V, V_PH_ = 5.2 V.

To verify that these MZMs can operate with this type of modulation, a measurement setup was mounted in the laboratory (see Figure 2a). A bias point controller similar to the one presented in [26] (but adapted to the characteristics of the LN86P–FC MZM) was implemented to ensure the stable operation of the modulator (Figure 2b). The frequency up-converted signal was generated by modulating a 1550 nm laser with a 30 GHz signal generated by a Gunn diode coupled to the MZM via a 90 degree waveguide hybrid (Figure 2c). The modulated signal was then measured using an optical spectrum analyzer (BOSA), which is the same one used in [26]. Figure 2d shows the modulated MZM output signal measured with the optical spectrum analyzer. These MZMs have three control voltages that determine the bias point of operation: V_RF1_, V_RF2_, and V_PH_. The bias point of (d) is determined using the method of setting the MZM for SSB-SC modulation, as defined by the commercial vendor. 

**Figure 3:**
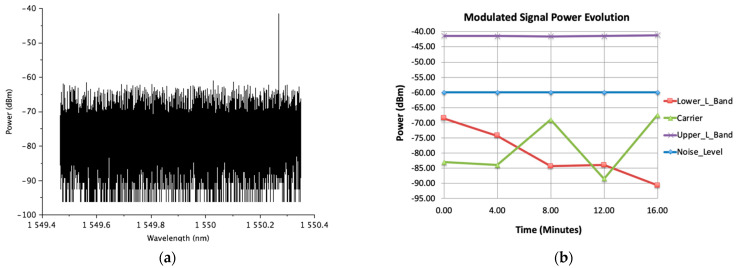
(**a**) Modulated signal measured with the optical spectrum analyzer for the MZM bias point: V_RF1_ = 4.9 V, V_RF2_ = 5.8 V, V_PH_ = 5.5 V; (**b**) time evolution of the modulated signal frequency components when the MZM is biased in the bias point of (**a**). The time evolution of the power levels at the frequencies of the carrier (green triangles), lower side band (red squares) and upper side band (purple crosses) are shown. For reference, the mean value of the noise floor maximum power level is also shown (blue diamonds).

Then, with small modifications of these voltages, one of the sidebands and the carrier fall below the signal noise level (see Figure 3a). Finally, in Figure 3b, it can be seen that the SSB–SC modulation is stable and maintained over time, thanks to the bias point controller.

From Figure 2d and Figure 3b, a carrier extinction ratio (ER) of more than 25 dB can be deduced. As can be seen in Section 4, this ER value allows the measurement of the microwave polarization with very good performance in terms of angle and degree (or efficiency) error.

As mentioned above, this kind of operation is highly recommended for the final polarimetry application, which works like an interferometer. However, the main problem we found for its practical application is the high cost of this kind of commercial MZM (see, e.g., [29]), considering that at least two MZMs per receiver would be needed in the most optimized polarimeter design [25]. 

To overcome this problem, in this work, we propose the use of MZMs fabricated using InP integrated technology, which can help reduce the price of the modulators, thus contributing to the viability of the proposed instrumentation.

## 3. InP-Integrated Technology MZMs

**Figure 4:**
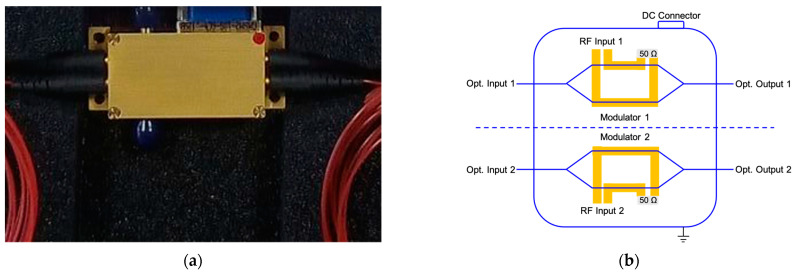
Chassis with two InP integrated MZM provided by HHI (**a**); device diagram showing a simplified layout of the two MZMs inside the chassis. The optical inputs and outputs, the RF inputs, and the DC connector situation are detailed following their physical disposition (**b**).

In this section, the use of integrated InP technology MZMs is proposed and justified, first in terms of the bandwidth that can be provided. Taking advantage of the integration capabilities of the technology, a new dual modulator design was mounted in a common package. These two integrated modulators were provided by HHI and tested in the laboratory. Figure 4 shows a photograph of the package containing the InP MZMs (a) and a schematic showing a simplified layout of the two MZMs inside the package (b). In terms of size, the advantage of this technology is clear if we compare the 42 × 23 mm^2^ size of this 2-unit package in Figure 4a with the 87 × 13 mm^2^ size of the commercial unit shown in Figure 2c (these values exclude the optical and RF connectors). This represents a size reduction factor greater than 2 in terms of area. In addition, it is important to note that although the package of Figure 4a was fabricated for a pair of MZMs, the internal InP chip contains twelve MZMs, but only two of them are connectorized. 

This was the first time HHI provided an integrated design with two operational MZMs on the same chip, so a new modulator design was developed just to meet our application integration requirements. 

**Figure 5:**
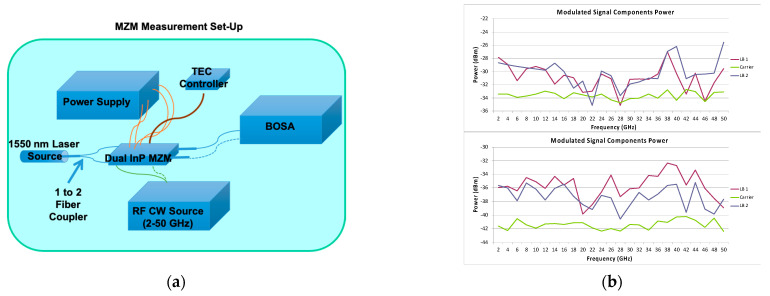
Laboratory characterization set-up scheme (**a**); the power of the modulated signal components, measured using the optical spectrum analyzer (BOSA) (**b**).

Figure 5a shows an schematic of the laboratory measurement setup used to test their true bandwidth. It can be seen that in this case, the MZM bias points were controlled by means of a TEC controller (model TTC001, 4W T–Cube TEC controller from Thorlabs), which has a similar effect as the bias point controllers mentioned in the previous section, but controls the internal temperature variations of the MZM instead of directly controlling the bias point of the modulators. A microwave signal generator was used to generate a continuous wave (CW) signal with a frequency swept from 2 to 50 GHz. This signal was used to modulate a 0 dBm-power 1550 nm wavelength laser in such a way that a modulated signal can be measured at the output of the MZMs using an optical spectrum analyzer (BOSA). These modulators do not provide the possibility of obtaining SSB–SC modulation, so instead, a dual-side band with suppressed carrier (DSB–SC) modulation is measured and used in the next section for the measurement of microwave polarization. In this sense, Figure 5b shows the power levels of the main modulated signal components, side bands (LB) 1 and 2, and the carrier, when sweeping the frequency of an RF input signal with 5 dBm power (2–50 GHz).

First, it can be observed that the bandwidth of the modulators reaches 50 GHz, although the nominal bandwidth of the InP technology provided by HHI is 40 GHz.

On the other hand, the bias points of both MZMs have been chosen to obtain an optimal carrier suppression degree within the total bandwidth. However, it can be observed that the carrier is only about 6 dB below the side bands, and not for all the frequencies. However, since these MZMs must be used with DSB–SC modulation, for our polarimetry application, we can take advantage of both sidebands using the polarimeter in direct imaging mode instead of interferometry mode, see [19]. In such a case, the power of both bands can be considered, thus providing a higher practical ER value (about 10 dB) considering the two sidebands and the carrier of the modulated signal. In fact, as we will see in the next section, these two modulators give good results (very similar to those of the commercial unit) when applied to the polarization measurement of a microwave signal.

## 4. Application to a Microwave Polarimeter Demonstrator

**Figure 6:**
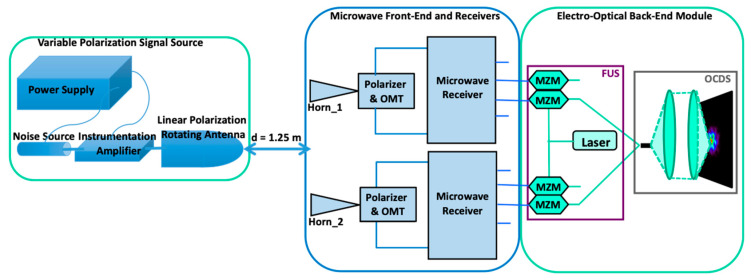
Sketch of the polarimeter demonstrator’s calibration test bench. The blue part corresponds to that implemented in microwave technology, while the green one is implemented by means of photonic/optical technology. Reproduced with permission from F. J. Casas, *Sensors*; published by MDPI, 2019 [19].

In this section, the proposed modulation technologies are applied to the microwave polarimeter presented in [19]. A sketch of the laboratory polarization measurement set-up is shown in Figure 6. The wideband source used in the setup is a variable polarization angle microwave signal source that takes advantage of a rotating antenna connected to a wideband amplifier and a noise source. It provides a 10–20 GHz bandwidth input signal that is 100% linearly polarized and has a variable (0–180 degrees) manually controlled polarization angle (see [19] for more details).

The type of measurements performed and the setup components have already been described in [19], and are the same as those used in [30] for polarization calibration. The only difference with the setup used in [19] is in the MZMs used to implement the frequency up-conversion stage (FUS), which for this work are the modulators previously shown plus an additional one from HHI, which shares the technology (InP) with those of Section 3. The use of a common setup facilitates the comparison of the polarization measurement results, since the observed differences will mainly be related to the use of one or the other type of modulator.

### 4.1. Polarization Measurement Results with LiNbO_3_ Commercial MZMs

**Figure 7:**
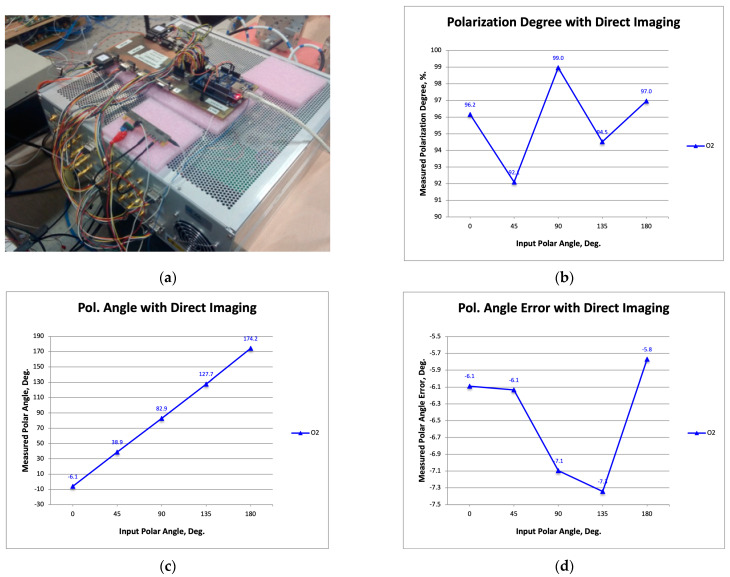
Photograph showing the rack with the polarimeter receivers and one commercial modulator, which is the same that was presented in Section 2 (**a**); the measured polarization degree (**b**), polarization angle (**c**), and its error defined as the difference between the measured polarization angle and the one from the signal source (**d**).

Figure 7a is a photograph showing the rack with the polarimeter receivers and a commercial modulator, which is the one presented in Section 2. Figure 7b–d show the measured polarization degree, the polarization angle, and its error (the difference between the measured angle and that of the signal source).

At this point, it is important to make a comparison with the previously published results (Figure 9 in [19]), which were obtained using limited-bandwidth commercial MZMs operating with DSB–SC modulation instead of the SSB–SC used in this work. 

The first important difference is that in [19], a polarization degree of about 50% was achieved, while here, a degree of about 95% was measured. This result is justified by the higher bandwidth of the commercial modulator used in this work (15 GHz) compared to the previous ones (10 GHz).

On the other hand, regarding the polarization angle error as a function of the input signal polar angle, a similar mean value is observed (around −6.5 degrees in this work and −6 degrees in [19]), while the amplitude of the error around the mean value is slightly smaller (1.5 degreed compared to 2.5 degrees in [19]). As shown in [30], the mean value offset can be easily corrected, while the error amplitude is a very important measure of the polarization calibration; therefore, in the following, we will compare it with the one obtained using the InP alternative modulators.

In summary, the use of the commercial modulator proposed in this work presents clear advantages compared to those used in previous works, except for factors such as the high cost of this type of modulator; this cost is mainly increased due to the higher bandwidth and the lack of integration capabilities within the LiNbO_3_ technology. 

### 4.2. Polarization Measurement Results with InP MZMs

**Figure 8:**
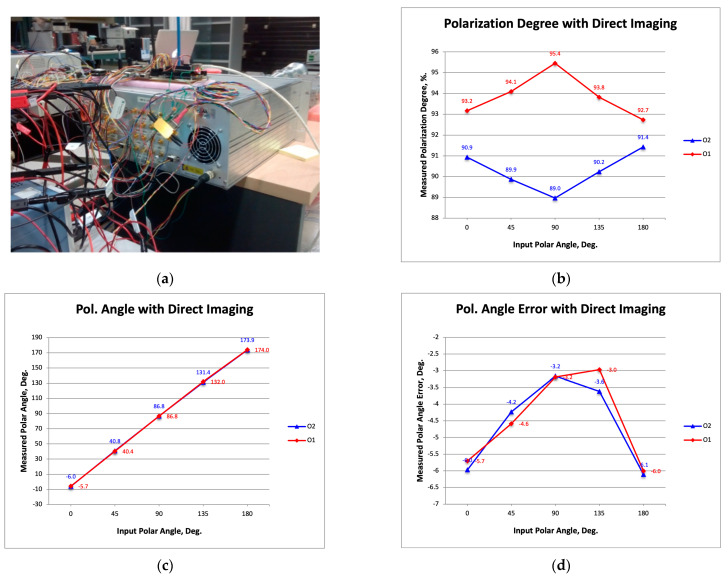
Photograph showing the rack with the polarimeter receivers and the InP modulators presented previously in Section 3 (**a**); the measured polarization degree (**b**), polarization angle (**c**), and its error defined as the difference between measured polarization angle and the one from the signal source (**d**).

Figure 8a is a photograph showing the rack with the polarimeter receivers and the InP modulators introduced in Section 3. Figure 8b–d show the measured degree of polarization, the angle of polarization, and its error (the difference between the measured angle and that of the signal source).

It is now possible to compare the results with those previously published [19] and with those shown in Figure 7. At the level of performance, the advantages of the InP modulators with respect to those used in previous works are similar to those provided by the commercial modulator of the present work. On the other hand, by comparing the results of Figure 7 and Figure 8, a similar degree of polarization can be observed (a little lower in the case of one of the InP modulators), while in terms of polarization angle error, a lower average value is observed (around −4.5 degrees of Figure 8d compared to the 6 degrees of Figure 7d). However, in terms of the polarization angle error amplitude, the InP MZMs provides a higher value (about 3 degrees compared to about 1.5 degrees), which could be initially attributed to the signal modulation type (DSB-SC of the InP MZMs, instead of SSB-SC of the commercial modulator). This last consideration is supported by the fact that in [19], a similar amplitude of the polar angle error (about 2.5 degrees) was shown with commercial modulators also using DSB-SC modulation. However, considering the novelty of the InP dual MZM design (this was the first time that HHI provided an integrated design with two MZMs), we decided to test the use of an additional InP modulator with a more well-founded design, just to avoid possible immaturity issues. 

**Figure 9:**
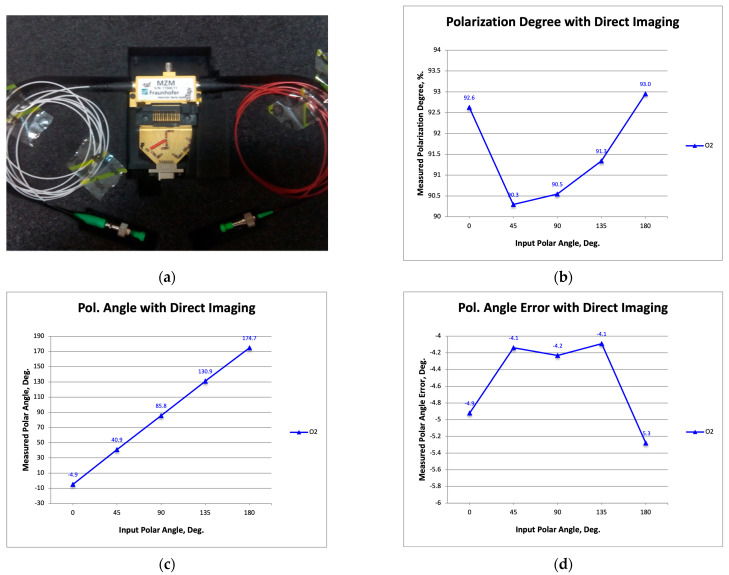
Photograph showing the InP additional modulator provided by HHI (**a**); the measured polarization degree (**b**), polarization angle (**c**), and its error defined as the difference between measured polarization angle and the one from the signal source (**d**).

This new modulator unit was also provided by HHI, and is shown in the photograph in Figure 9a. It is important to note that the size of this modulator package is very similar to that of the dual MZMs. The measured degree of polarization shown in Figure 9b is similar to that provided by the polarimeter with the dual modulator design. Additionally, the measured polar angle error shown in Figure 9d has a similar average value (about −4.7 degrees). However, the amplitude of the error (about 1.2 degrees) is even smaller than that of the commercial modulator, so it seems that the novelty of the integrated dual modulator design has an effect on this aspect of the polar angle error. 

From this result, it can be expected that with additional effort to provide a more mature dual modulator design, the amplitude level of the polar angle error could be reduced to values such as the one shown in Figure 9d. 

## 5. Conclusions

This paper presents a study of the performance of different Mach–Zehnder modulation technologies and their application to microwave heterodyne polarimeters based on the use of an NIR frequency up-conversion stage. Commercial MZMs have so far been implemented using LiNbO_3_ technology, which does not allow integration for the implementation of the frequency up-conversion stages. On the other hand, we have also tested the use of an alternative technology based on InP, which allows integration in the fabrication processes, while offering potential advantages in terms of bandwidth, cost and size reduction. These aspects are very interesting for broadband applications such as microwave instrumentation for the study of the cosmic microwave background (CMB). 

We have shown the modulation performances of different MZMs, including a commercial unit with a higher bandwidth (15 GHz) than in previous works and SSB–SC modulation capabilities, and another two InP integrated units (a dual design and a second single modulator) provided by HHI. In the first part of the work, the good performance of the commercial modulator unit has been shown in terms of obtaining an SSB–SC modulation with an ER value of about 25 dB, which is particularly interesting for its application in synthesized imaging interferometers. The large bandwidth coverage (DC-50 GHz) of the InP modulators with practical ER values of about 10 dB was also demonstrated by measuring DSB-SC-modulated signals. 

In the second part of the paper, the modulators were coupled to a microwave polarimeter demonstrator presented in previous works to compare the polarization measurement performances corresponding to each of the MZM options. It was shown that the commercial modulator was able to provide optimal polarization percentage levels with a lower amplitude of the polarization angle error. On the other hand, despite their lower ER values, InP devices have shown similar performances in terms of polarization systematic errors, but have also shown advantages provided by their wide bandwidth and technology integration capabilities, which will have an impact via size and cost reduction. In fact, several inputs from the foundry allow us to expect a cost reduction of about a factor of 10 compared to commercial LiNbO_3_ technology (and considering applications with hundreds of receivers, as can be the case in CMB instrumentation). For all these reasons, we believe that the use of integrated InP modulators is an excellent option for broadband microwave astronomy applications, such as ground-based CMB polarization experiments.

## Data Availability

Data available on request due to privacy restrictions. The data presented in this study are available on request from the corresponding author. The data are not publicly available due to privacy reasons.

## References

[1] Penzias A.A., Wilson R.W. (1965). A Measurement of Excess Antenna Temperature at 4080 Mc/s. Astrophys. J..

[2] Alpher R.A., Bethe H., Gamow G. (1948). The Origin of Chemical Elements. Phys. Rev..

[3] Buder I. (2010). Q/U Imaging Experiment (QUIET): A ground-based probe of cosmic microwave background polarization. Proceedings of the Millimeter, Submillimeter, and Far-Infrared Detectors and Instrumentation for Astronomy V.

[4] Barkats D., Aikin R., Bischoff C., Buder I., Kaufman J.P., Keating B.G., Kovac J.M., Su M., Ade P.A.R., Battle J.O. (2014). Degree-Scale Cosmic Microwave Background Polarization Measurements from three years of BICEP1 data. Astrophys. J. Lett..

[5] Ade P.A.R., Ahmed Z., Aikin R.W., Alexander K.D., Barkats D., Benton S.J., Bischoff C.A., Bock J.J., BICEP2 Collaboration, Keck Array Collaboration (2018). Constraints on Primordial Gravitational Waves using Planck, WMAP, and new BICEP2/Keck Observations through the 2015 season. Phys. Rev. Lett..

[6] Leitch E.M., Kovac J.M., Halverson N.W., Carlstrom J.E., Pryke C., Smith M.W.E. (2005). Degree Angular Scale Interferometer 3 Year Cosmic Microwave Background Polarization Results. Astrophys. J. Lett..

[7] Ade P.A.R., Akiba Y., Anthony A.E., Arnold K., Atlas M., Barron D., Boettger D., Borrill J., Chapman S., Chinone Y. (2014). Measurement of the Cosmic Microwave Polarization lensing power spectrum with the POLARBEAR experiment. Phys. Rev. Lett..

[8] Hanson D., Hoover S., Crites A., Ade P.A.R., Aird K.A., Austermann J.E., Beall J.A., Bender A.N., Benson B.A., Bleem L.E. (2013). Detection of B-mode polarization in the Cosmic Microwave Background with data from the South Pole Telescope. Phys. Rev. Lett..

[9] Masi S., Ade P.A., Bock J.J., Bond J.R., Borrill J., Boscaleri A., Cabella P., Contaldi C.R., Crill B.P., de Bernardis P. (2006). Instrument, method, brightness and polarization maps from the 2003 flight of BOOMERanG. Astron. Astrophys..

[10] Rabii B., Winant C.D., Collins J.S., Lee A.T., Richards P.L., Abroe M.E., Hanany S., Johnson B.R., Ade P., Balbi A. (2006). MAXIMA: A balloon-borne cosmic microwave background anisotropy experiment. Rev. Sci. Instrum..

[11] Bennett C.L., Banday A.J., Gorski K.M., Hinshaw G., Jackson P., Keegstra P., Kogut A., Smoot G.F., Wilkinson D.T., Wright E.L. (1996). Four-Year COBE DMR Cosmic Microwave Background Observations: Maps and Basic Results. Astrophys. J. Lett..

[12] Bennett C.L., Larson D., Weiland J.L., Jarosik N., Hinshaw G., Odegard N., Smith K.M., Hill R.S., Gold B., Halpern M. (2013). Nine-year Wilkinson Microwave Anisotropy Probe (WMAP) Observations: Final Maps and Results. Astrophys. J. Suppl. Ser..

[13] Akrami Y., Arroja F., Ashdown M., Aumont J., Baccigalupi C., Ballardini M., Banday A., Barreiro R., Bartolo N., Planck Collaboration (2020). Planck Collaboration. Planck 2018 results. I. Overview, and the cosmological legacy of Planck. Astron. Astrophys..

[14] Planck Collaboration (2020). Planck 2018 results—VI. Cosmological parameters. Astron. Astrophys..

[15] Tristram M., Banday A.J., Górski K.M., Keskitalo R., Lawrence C.R., Andersen K.J., Barreiro R.B., Borrill J., Colombo L.P.L., Eriksen H.K. (2022). Improved limits on the tensor-to-scalar ratio using BICEP and Planck. Phys. Rev. D.

[16] Abazajian K., Crites A., Hui H., Moncelsi L., Schillaci A., Bock J. (2019). CMB-S4 Decadal Survey APC White Paper. arXiv.

[17] Sugai H., Ade P.A.R., Akiba Y., Alonso D., Arnold K., Aumont J., Austermann J., Baccigalupi C., Banday A.J., Banerji R. (2020). Updated Design of the CMB Polarization Experiment Satellite LiteBIRD. J. Low Temp. Phys..

[18] Hanany S., Alvarez M., Artis E., Ashton P., Aumont J., Aurlien R., Banerji R., Barreiro R.B., Bartlett J.G., Basak S. (2019). PICO: Probe of Inflation and Cosmic Origins. arXiv.

[19] Casas F.J., Ortiz D., Aja D., de la Fuente L., Artal E., Ruiz R., Mirapeix J.M. (2019). A microwave polarimeter demonstrator for astronomy with near-infra-red up-conversion for optical correlation and detection. Sensors.

[20] Casas F.J., Ortiz D., Villa E., Cano J.L., Cagigas J., Pérez A.R., Aja B., Terán J.V., de la Fuente L., Artal E. (2015). The Thirty Gigahertz Instrument Receiver for the QUIJOTE Experiment: Preliminary Polarization Measurements and Systematic-Error Analysis. Sensors.

[21] Hamilton J.-C., Mousset L., Battistelli E., De Bernardis P., Bigot-Sazy M.-A., Chanial P., Charlassier R., D’Alessandro G., De Petris M., Lerena M.G. (2022). Qubic i: Overview and science program. J. Cosmol. Astropart. Phys..

[22] Villa E., Cano J.L., Cagigas J., Ortiz D., Casas F.J., Pérez A.R., Aja B., Terán J.V., de la Fuente L., Artal E. (2015). The thirty-gigahertz instrument receiver for the Q-U-I Joint Tenerife experiment: Concept and experimental results. Rev. Sci. Instrum..

[23] Addamo G., Ade PA R., Baccigalupi C., Baldini A.M., Battaglia P.M., Battistelli E.S., Baù A., de Bernardis P., Bersanelli M., The LSPE collaboration (2021). The large scale polarization explorer (LSPE) for CMB measurements: Performance forecast. J. Cosmol. Astropart. Phys..

[24] Collet E. (2005). Field Guide to Polarization.

[25] Pascual-Cisneros G., Casas F.J., Vielva P. (2023). Optimization of a microwave polarimeter for astronomy with optical correlation and detection. Sensors.

[26] Ortiz D., Casas F.J., Ruiz-Lombera R., Mirapeix J. (2017). Electro-optic correlator for large-format microwave interferometry: Up-conversion and correlation stages performance analysis. Rev. Sci. Instrum..

[27] Rubiño-Martín J.A., Guidi F., Génova-Santos R.T., Harper S.E., Herranz D., Hoyland R.J., Lasenby A.N., Poidevin F., Rebolo R., Ruiz-Granados B. (2023). QUIJOTE scientific results—IV. A northern sky survey in intensity and polarization at 10–20 GHz with the multifrequency instrument. Mon. Not. R. Astron. Soc..

[28] Thorlabs LN86S-FC MZM. https://www.thorlabs.com/thorproduct.cfm?partnumber=LN86S-FC&pn=LN86S-FC.

[29] Thorlabs LNQ4314 MZM. https://www.thorlabs.com/thorproduct.cfm?partnumber=LNQ4314.

[30] Casas F.J., Vielva P., Barreiro R.B., Martínez-González E., Pascual-Cisneros G. (2022). Polarization Calibration of a Microwave Polar- imeter with Near-Infrared Up-Conversion for Optical Correlation and Detection. Sensors.

